# Demographic and geographic hotspots of Alzheimer’s disease and pneumonia-related mortality among older adults in the United States: a retrospective analysis

**DOI:** 10.1186/s40001-026-03915-x

**Published:** 2026-01-24

**Authors:** Muhammad Shaheer Bin Faheem, Shamikha Cheema, Muhammad Raza, Anushree Rai, Ahmed Abdullah, Tehreem Asghar, Syed Tawassul Hassan, Faraz Arshad, Hassan Ijaz Cheema, Muhammad Faique, Sumaya Samadi

**Affiliations:** 1 Karachi Institute of Medical Sciences, KIMS, Karachi, Sindh Pakistan; 2 King Edward Medical University, Lahore, Pakistan; 3 Jinnah Sindh Medical University, Karachi, Pakistan; 4 Chhattisgarh Institute of Medical Sciences, Bilaspur, Chhattisgarh India; 5 Khyber Medical College, Peshawar, Pakistan; 6 Akhtar Saeed Medical & Dental College, Lahore, Pakistan; 7 Karachi Medical and Dental College, KMDC, Karachi, Sindh Pakistan; 8 Shaikh Zayed Hospital, Lahore, Pakistan; 9 Kabul University of Medical Sciences “Abu Ali Ibn Sina”, Kabul, Afghanistan

**Keywords:** Alzheimer’s disease, Pneumonia, Mortality rates, United States, CDC-WONDER

## Abstract

**Background:**

Older patients with Alzheimer’s disease (AD) are more susceptible to pneumonia because of their limited mobility, malnourishment, and difficulty swallowing. This study highlights demographic and geographic while examining AD and pneumonia-related mortality rates among older adults in the United States (U.S.) between 1999 and 2023.

**Methods:**

Retrospective cohort research utilized mortality data from the Centre for Disease Control and Prevention’s Wide-Ranging Online Data for Epidemiologic Research (CDC-WONDER). Demographic factors (age, gender, race/ethnicity) and geographic classifications (urbanization, regions, states) were examined during the analysis. We used joinpoint regression and standardization techniques to determine age-adjusted mortality rates (AAMR) and annual percentage changes (APC).

**Results:**

A total of 202,678 AD and pneumonia-related deaths were reported. Overall AAMR declined significantly from 1999 to 2023 (APC − 6.65; P < 0.05). From demographics males (22), non-Hispanic (NH) Whites (20.8), individuals aged 85 + years (98.3) had the highest mortality rates. Geographically, residents of non-metropolitan (25.6) and west regions (26.9) represented peak rates.

**Conclusion:**

We observed notable demographic and geographic disparities in AD and pneumonia associated mortality, the burden of which is higher among males, NH Whites and those residing in non-metropolitan regions and therefore we emphasize the need of targeted interventions and equitable resource allocation to reduce this burden especially among high-risk populations.

**Graphical Abstract:**

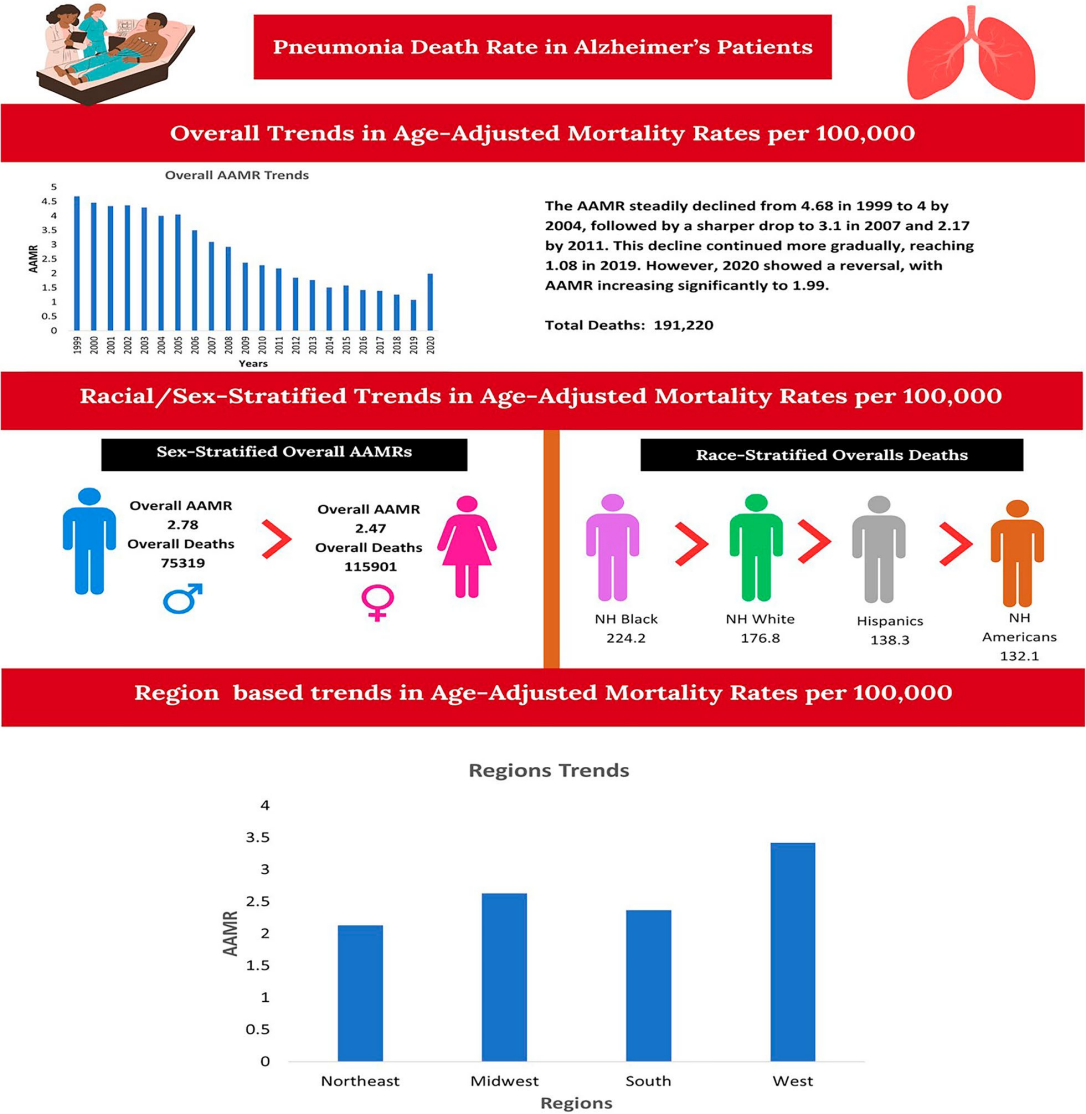

## Introduction

Pneumonia remains a leading cause of death and hospitalization among older adults in the United States (U.S.), marking itself a major public health concern, with hospitalization rates ranging from 5500 to 7000 per 100,000 in adults aged ≥ 65 years and rising significantly to over 16,000 per 100,000 in those aged 80 years and above [1]. However, from 1999 to 2022, the age-adjusted mortality rates (AAMRs) among individuals aged ≥ 85 years have declined by 68% and despite this, pneumonia patients continue to bear the highest risk of mortality and a need for preventive approaches [2]. Among the older population, those with Alzheimer’s disease (AD) face an even greater risk. The risk of pneumonia-associated mortality increased by two-fold with dementia, while the hospitalization risk increased by 70% [3, 4]. Pneumonia commonly accounts for 25–30% of deaths in AD patients and is identified as an immediate or terminal cause of death [5].

Multiple converging mechanisms in AD have been known to contribute to pneumonia risk, including dysphagia, aspiration, and impaired immune function [6, 7]. The impaired gag and cough reflexes, reduced mobility, immune dysfunction, malnutrition, and the use of sedative medications can increase pneumonia mortality [6, 7]. Aspiration pneumonia itself is a leading cause of death in late-stage AD [6]. Despite the significance of these combined pathologies, no study has comprehensively evaluated AD and pneumonia-associated mortality trends and disparities in older individuals, while previous studies have described pneumonia and AD mortality separately; they overlooked their combined epidemiologic pattern and existing disparities across demographics and geographics [8]. Recognizing this gap, our study analyzes the trends and discrepancies of pneumonia and AD-associated mortality across age, gender, urbanization, regions, states, and places of death in the U.S. from 1999 to 2023. The findings will help determine the most vulnerable population, which is useful for policymakers to target those demographics that are most affected by this disease.

## Methods

### Study design and population

A retrospective analysis of death records listing pneumonia and AD was done from 1999 to 2023 in the U.S. using the CDC WONDER multiple cause of death database, which is a publicly available system that compiles the death certificate data collected by the National Center for Health Statistics [9]. Only those death certificates were included in which each listed both pneumonia and AD either as the underlying or contributing cause of death. These records covered all 50 states and the District of Columbia and were identified through International Classification of Diseases, 10th Revision, Clinical Modification (ICD-10-CM) codes: J12-J18 (viral, bacterial, and unspecified pneumonia) and G30 (AD). These code selections were based on the recent nationwide epidemiological analysis of pneumonia/AD mortality trends utilizing the same database [10–12]. Adults aged ≥ 65 years were included in our study, while those younger than the defined age group were excluded due to low counts and unstable rate estimates. Further, the open-access nature of the database obviated the need for institutional review board approval. STROBE guidelines were strictly followed [13].

### Data extraction

Datasets were stratified across demographics (age, sex, race/ethnicity, and place of death) and geographics (urbanization level, census regions, and states). Racial and ethnic groups were defined as non-Hispanic (NH: American Indian/Alaska Native, White, African American, Asian/Pacific Islander) and Hispanics. Urbanization level classifications followed the 2013 NCHS Urban–Rural Classification Scheme for Counties and included metropolitan and non-metropolitan areas [14]. Northeast, South, Midwest, and West were the distinct regional categories, while medical facilities like hospitals, care institutions, and home settings were the reporting places of death.

### Statistical analysis

Crude mortality rates (CMRs) were determined by dividing the total number of deaths from pneumonia and AD by the total population in that year. AAMR were calculated per 100,000 by age-adjusting the population using the 2000 U.S. census data to reflect changes in the age distribution, and confidence intervals (CI) were estimated under the assumption of a Poisson distribution [15]. After calculating the AAMR, they were analyzed using the joinpoint regression software to estimate the temporal trends in mortality [16]. The program calculates the significant changes in the AAMR, using linear regression analysis, and graphs these changes by connecting the joinpoints. Furthermore, the upward and downward trends were determined logistically by slopes that were significantly different from zero. These trends are presented as the annual percent changes (APC) in mortality rates for AAMR over the course of time, with statistically significant differences at the p < 0.05 level using a two-sided t-test. Furthermore, the data determined that Bayesian information weighted criteria were utilized to determine the optimal model, which allowed up to four joinpoints.

## Results

**Fig. 1:**
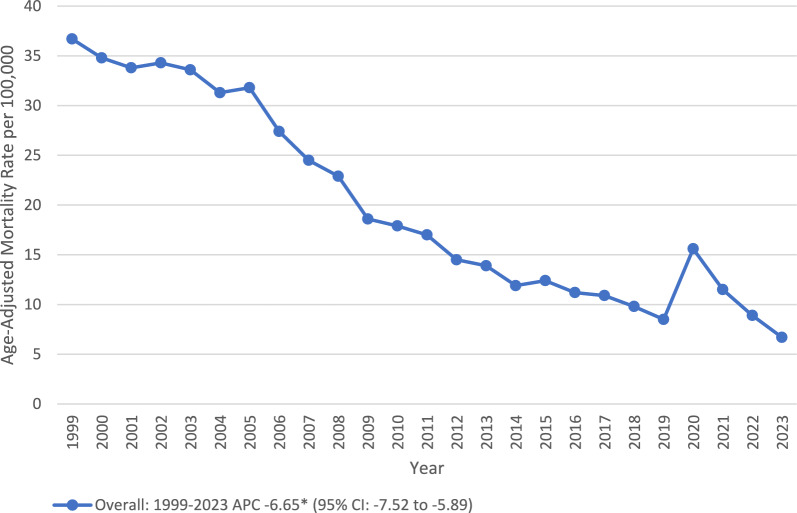
Overall, trends in Alzheimer’s disease and pneumonia-related age-adjusted mortality rates per 100,000 among older patients in the United States, 1999 to 2023. APC = Annual Percentage Change, CI = Confidence Interval. *Indicates that the Annual Percentage Change (APC) is significantly different from zero at α = 0.05

A total of 202,678 deaths occurred among individuals with pneumonia and AD. The AAMR declined significantly from 1999 to 2023 (APC: − 6.65*; 95% CI − 7.52 to − 5.89). Females (60.51%) experienced higher deaths than males (39.49%). NH White (86.63%) made the highest proportion of overall mortalities, followed by NH Black or African American (5.57%), Hispanic or Latino (5.16%), and NH Asian or Pacific Islander (2.18%). Among the older adults, those who were aged 85 + (62.48%) reported the highest mortality. Nursing home (50%) reported the highest deaths, followed by medical facility (31%), decedent’s home (8%), and hospice facility (3%). Deaths were more prevalent in metropolitan (77.47%) than in non-metropolitan areas (22.53%), and a larger number of mortalities were recorded in the South region (32.33%), followed by West (27.74%), Midwest (23.21%), and Northeast (16.73%) (Fig. 1) (Table 1).

**Table 1:** Demographic Characteristics of patient’s deaths due to Alzheimer’s disease and Pneumonia among older patients in the United States from 1999–2023

Variable	Deaths (% total deaths)	Total average AAMR (95% CI) per 100,000
Overall population	202,678 (100)	20 (19.6–20.4)
Gender		
Male	80,040 (39.49)	22 (21.2–22.7)
Female	122,638 (60.51)	18.8 (18.3–19.3)
Race/ethnicity		
Asian or Pacific Islander	4421 (2.18)	13.2 (11.2–15.3)
Black or African American	11,291(5.57)	14.4 (13.1–15.7)
White	175,574 (86.63)	20.8 (20.3–21.3)
Hispanic or Latino	10,463 (5.16)	17.3 (15.6–19)
Age^a^		Total Average CMR (95% CI) per 100,000
65–74 years	12,037 (5.94)	2.2 (2–2.4)
75–84 years	64,017 (31.59)	19.1 (18.4–19.8)
85 + years	126,624 (62.48)	98.3 (95.7–100.9)
Census region		Total Average AAMR (95% CI) per 100,000
Northeast	33,901 (16.73)	16 (15.2–16.8)
Midwest	47,039 (23.21)	19.7 (18.9–20.6)
South	65,516 (32.33)	18.6 (17.9–19.3)
West	56,222 (27.74)	26.9 (25.8–27.9)
Urbanization		
Metropolitan areas	146,380 (77.47)	19.2 (19.1–19.3)
Non-metropolitan areas	42,565 (22.53)	25.6 (25.4–25.9)
Place of death^c^		
Medical facilities	63,430 (31)	Not applicable
Decedent’s home	15,935 (8)	Not applicable
Hospice facility	5608 (3)	Not applicable
Nursing home/long term care facility	101,550 (50)	Not applicable
Other	6693 (3)	Not applicable

Age-specific values represent Crude Mortality Rates (CMRs) rather than Age-Adjusted Mortality Rates (AAMRs), as adjustment cannot be applied within individual age strata.

Age Adjusted Mortality Rate (AAMR) does not apply to the Place of Death.

APC indicates Annual Percent Change *P < 0.05

### Sex

**Fig. 2:**
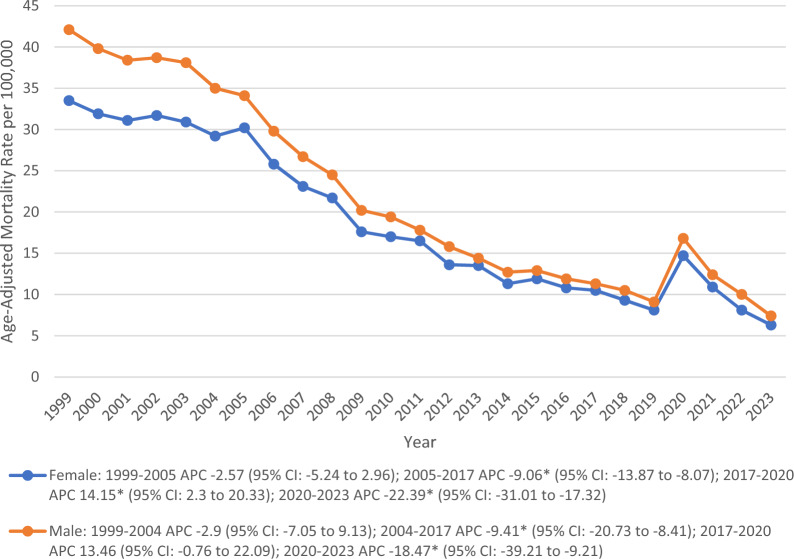
Trends in Alzheimer’s disease and pneumonia-related age-adjusted mortality rates per 100,000, stratified by sex among older patients in the United States, 1999 to 2023. APC = Annual Percentage Change, CI = Confidence Interval. *Indicates that the Annual Percentage Change (APC) is significantly different from zero at α = 0.05

The total average AAMR in males (22) was higher than that of females (18.8). Females showed a constant decline in AAMR from 1999 to 2004, followed by a sharp decrease through 2017 (APC − 9.06*; 95% CI − 13.87 to − 8.07). Following that a stark rise was observed until 2020 (APC 14.15*; 95% CI 2.3 to 20.33) before declining again till 2023 (APC − 22.39*; 95% CI − 31.01 to − 17.32). However, in males, the AAMR began to decline significantly after 2004 till 2017 (APC − 9.41*; 95% CI − 20.73 to − 8.41), followed by a steady rise through 2020 and declined significantly thereafter (APC − 18.47*; 95% CI − 39.21 to − 9.21) (Fig. 2) (Table 1).

### Race

**Fig. 3:**
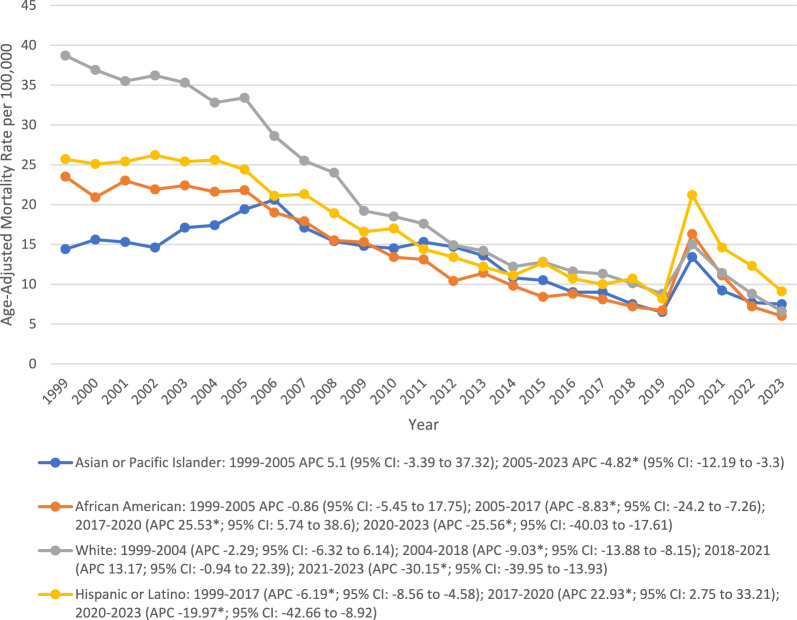
Trends in Alzheimer’s disease and pneumonia-related age-adjusted mortality rates per 100,000, stratified by race and ethnicity among older patients in the United States, 1999 to 2023. APC = Annual Percentage Change, CI = Confidence Interval. *Indicates that the Annual Percentage Change (APC) is significantly different from zero at α = 0.05

NH White (20.8) represented the highest average rate (20.8), followed by Hispanic (17.3), NH Black (14.4), and NH Asian (13.2). NH White experienced a significant decline in AAMR between 2004 and 2018 (APC − 9.03*; 95% CI − 13.88 to − 8.15), followed by a steady rise until 2021, and showed a significant decline (APC − 30.15*; 95% CI − 39.95 to − 13.93) thereafter. NH African after a significant decline between 2005 and 2017 showed a sharp rise until 2020, followed by a significant decrease through 2023 with associated APCs of − 8.83* (95% CI − 24.2 to − 7.26), 25.53* (95% CI 5.74 to 38.6), and − 25.56* (95% CI − 40.03 to − 17.61), respectively. A similar upsurge in mortality rate was observed in Hispanics between 2017 and 2020 (APC 22.93*; 95% CI 2.75 to 33.21) before declining again until 2023. However, NH Asians experienced a constant increase until 2005, followed by a sharp decrease through 2023 (APC − 19.97*; 95% CI − 42.66 to − 8.92) (Fig. 3) (Table 1).

### Age

**Fig. 4:**
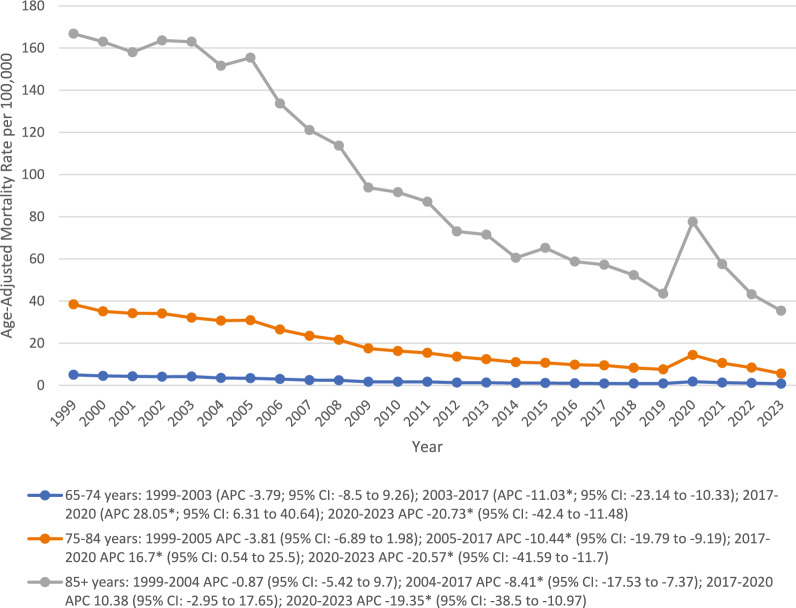
Trends in Alzheimer’s disease and pneumonia-related age-adjusted mortality rates per 100,000, stratified by age groups among older patients in the United States, 1999 to 2023. APC = Annual Percentage Change, CI = Confidence Interval. *Indicates that the Annual Percentage Change (APC) is significantly different from zero at α = 0.05

Overall, the average CMR in individuals aged 85 + (98.3) was ninefold higher than that of those aged 75–84 (19.1) and 65–74 (2.2) years. All the age groups showed a steady decline in CMRs in the early 2000s, which gained significance through 2017. Following 2017, a rise in CMR was observed across all the age groups until 2020, being significant in 65–74 (APC 28.05*; 95% CI 6.31 to 40.64) and 75–84 year (APC 16.7*; 95% CI 0.54 to 25.5). After that, a significant reduction in rates was observed through 2023 (Fig. 4) (Table 1).

### Geographics

**Fig. 5:**
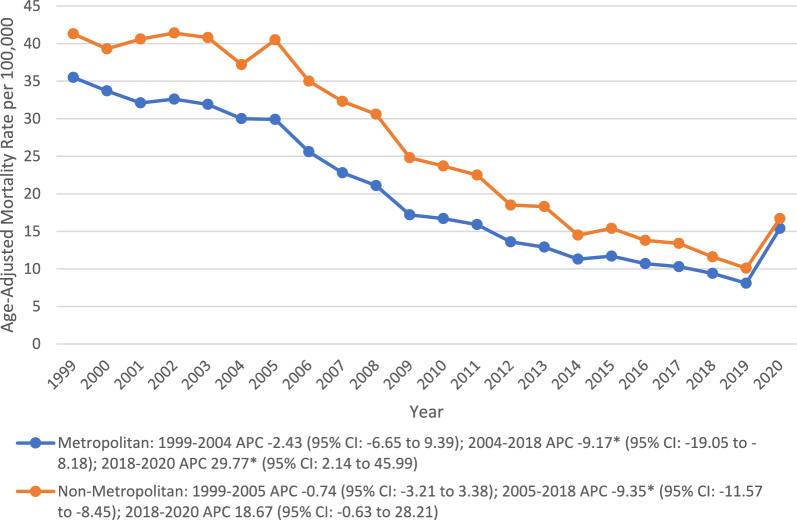
Trends in Alzheimer’s disease and pneumonia-related age-adjusted mortality rates per 100,000, stratified by urbanization among older patients in the United States, 1999 to 2020.APC = Annual Percentage Change, CI = Confidence Interval. *Indicates that the Annual Percentage Change (APC) is significantly different from zero at α = 0.05

Significant disparities were observed across different geographical subgroups in the U.S., with states (top 90th percentile: California, North Dakota, South Dakota, Washington, and Vermont) having 3 times the AAMR of states (Nevada, Florida, New York, Delaware, and New Jersey) in the lower 10th percentile. Region-wise peak rates were observed at the West (26.9), followed by the Midwest (19.7), the South (18.6), and the Northeast (16). Further, residents of non-metropolitan areas (25.6) experienced higher AAMR than the metropolitan population (19.2), with both showing steady declines in the early 2000s, which gained significance through 2018 (APC: metropolitan: − 9.17*; 95% CI − 19.05 to − 8.18; non-metropolitan: − 9.35*; 95% CI − 11.57 to − 8.45). After that, the rates increased significantly in metropolitan areas (APC 29.77*; 95% CI 2.14 to 45.99) and showed a steady rise in non-metropolitan regions through 2020 (Table 2) (Fig. 5).

**Table 2:** Pneumonia and Alzheimer’s Disease-related Age-Adjusted mortality Rates per 100,000, Stratified by States in the United States, 1999 to 2020

State	Age adjusted rate	Percentile (%)
Nevada	7.9 (7.2–8.6)	0
Florida	9.3 (9–9.5)	2
New York	14.2 (13.9–14.5)	4
Delaware	14.4 (13–15.8)	6
New Jersey	14.6 (14.2–15.1)	8
Pennsylvania	14.8 (14.5–15.2)	10
Maryland	15.2 (14.6–15.8)	12
Louisiana	15.4 (14.7–16.1)	14
New Mexico	15.6 (14.6–16.6)	16
Virginia	15.9 (15.4–16.4)	18
Alaska	16.2 (13.5–18.8)	20
Wisconsin	16.8 (16.2–17.4)	22
Arizona	17.1 (16.5–17.7)	24
Hawaii	17.1 (16–18.3)	24
Michigan	17.1 (16.6–17.6)	24
Illinois	17.4 (17–17.8)	30
Connecticut	18.8 (18–19.5)	32
District of Columbia	19 (16.9–21.1)	34
Mississippi	19.4 (18.4–20.3)	36
Kansas	19.7 (18.8–20.6)	38
Utah	19.7 (18.5–20.9)	38
Colorado	19.9 (19.1–20.7)	42
Montana	20 (18.5–21.5)	44
Georgia	20.4 (19.8–21)	46
Minnesota	20.4 (19.7–21.1)	46
Texas	20.7 (20.3–21.1)	50
Idaho	21.1 (19.8–22.5)	52
Rhode Island	21.2 (19.8–22.7)	54
Oregon	21.4 (20.6–22.2)	56
Missouri	21.8 (21.1–22.4)	58
Arkansas	22.4 (21.4–23.3)	60
South Carolina	22.4 (21.6–23.2)	60
Massachusetts	22.5 (21.9–23.1)	64
Alabama	22.6 (21.8–23.4)	66
Indiana	22.6 (21.9–23.3)	66
Ohio	22.9 (22.4–23.4)	70
Nebraska	23.2 (22–24.5)	72
Maine	23.9 (22.6–25.3)	74
New Hampshire	24.1 (22.6–25.6)	76
Iowa	24.3 (23.4–25.2)	78
Oklahoma	24.3 (23.4–25.2)	78
North Carolina	24.9 (24.3–25.5)	82
Wyoming	25.8 (23.3–28.4)	84
West Virginia	27.1 (25.8–28.4)	86
Kentucky	28.7 (27.7–29.6)	88
Tennessee	28.8 (28–29.5)	90
California	31.1 (30.7–31.4)	92
North Dakota	34.6 (32.4–36.9)	94
South Dakota	37.1 (34.9–39.3)	96
Washington	39.1 (38.2–39.9)	98
Vermont	40 (37.3–42.7)	100

## Discussion

Our study assesses the long-term mortality trends related to pneumonia and AD in the U.S. from 1999 to 2023. The purpose is to demonstrate how the concomitant diseases’ burdens have evolved over time and are differentially distributed by demographic and geographic location. The results indicate that the interrelationship of progressive cognitive decline, increased respiratory risk, and access to health care has contributed to the differential mortality outcomes for persons with AD and pneumonia. Mechanisms like weakened immune system, persistent inflammation, cognitive impairment, comorbidities, and direct action of the pathogen on the brain compound to form a vicious circle of vulnerability, with infections exacerbating cognitive impairment and vice versa, thereby enhancing the susceptibility to pneumonia in AD disease patients [17].

The overall decrease in AAMR for older adults from the early to mid-2000s can possibly be due to improved diagnosis and treatment of the elderly population, improved vaccination coverage, and improved treatment of respiratory infections [18]. However, the spike in AAMR between 2018 and 2020 mirrors the COVID-19 Pandemic time frame and the resultant overwhelming condition of the U.S. healthcare system and a significant increase in respiratory-related complications among all age groups [8, 19]. Therefore, it appears that the COVID-19 Pandemic increased the risk of pneumonia in older adults, particularly those with cognitive impairments, dysphagia, and/or decreased ambulation, which are the characteristics of advanced AD [8, 19]. However, the post-COVID decline in AAMRs may be an indication of a recovery period in the delivery of medical care and preventive practices; however, there are still many disparate trends among various subgroups. Recently, Yao et al. (2024) in their meta-analysis highlighted the differences between clinical and pathological findings, showing that the autopsy-confirmed pneumonia mortality in dementia (56.14%) is significantly higher than that reported on death certificates (16.12%). However, only 0.65% of the deaths were confirmed by autopsy in our data, which likely reflects the conservative death-certificate estimates described by Yao et al., potentially underestimating the true pathological burden of pneumonia in this population [20].

The observed sex-specific differences are of utmost importance because, even though women made up a larger percentage of total deaths, men had a higher AAMR. This difference is likely a result of the demographics of the population; because women live longer than men, they represent a larger portion of the older adult population with AD, and therefore make more total deaths; however, men have a significantly higher risk of death than women when looking at their age-adjusted risk [21, 22]. In addition, behavioral factors that make older men more susceptible to pneumonia, such as a higher incidence of smoking, smoking-related chronic lung disease, poor nutritional status, frailty, and multiple comorbidities, likely contribute to these findings [23]. Therefore, it’s imperative to develop both male and female-specific prevention programs focusing on modifiable risk factors for men, such as smoking, while also providing long-term care support to older women with AD.

Further, there were continued racial/ethnic differences observed throughout the study, with NH Whites recording the highest AAMR compared to other racial and ethnic groups. Our findings align with the existing literature, which has reported a high AAMR in the NH White population [24, 25]. These inequalities are an intricate interaction between genetic factors, socioeconomic factors, and healthcare access factors [24, 25]. Moreover, the increased mortality rates among these subgroups during the years prior to the pandemic (2017–2020) may have been due to limited access to healthcare and possibly poor vaccine acceptance or poor treatment of early pneumonia [26]. The incidence of pneumococcal and influenza vaccination is consistently lower in NH African American and Hispanic adults than for NH White adults, particularly in the older age groups, driving the concerning mortality spikes in these minority populations [26]. Further, this paradox, where the NH White population having better healthcare access experiences higher mortality, is likely attributable to survival bias and competing risks [25, 27]. NH Whites are historically recorded to have higher life expectancies compared to NH African American and Hispanic populations, who face higher rates of premature mortality from other conditions. Consequently, the risk of AD-associated pneumonia mortality is high in NH Whites as a larger proportion of this population survives to the advanced ages (≥ 85 years) where AD prevalence peaks [25, 27]. Although the CDC WONDER database did not allow for the extraction of specific socioeconomic data, these patterns suggest that while survival bias drives high cumulative rates in NH Whites, systemic inequities continue to exacerbate acute respiratory burdens in minority populations. [3].

Age is the most important predictor of pneumonia and AD-associated mortality, and the risk increases by 5% for each additional year of age [28]. The exponential increase in CMR noted in persons aged ≥ 85 years emphasizes the cumulative effects of frailty, compromised immunocompetency, and dysphagia [29]. Individuals with severe AD have frequent episodes of aspiration that may remain un-documented and therefore are the leading cause of death in patients who are at the final stages of their disease and thus the importance of timely swallowing evaluations, nutritional management, and consideration of palliative care has been emphasized in order to lessen the incidence of pneumonia-related complications associated with late-stage disease [6, 30].

Geographic variability within states adds more to our knowledge of these mortality patterns. States with the highest percentiles for AAMR (California, North Dakota, South Dakota, Washington, and Vermont) were about three times as high as states at the bottom 10th percentile (Nevada, Florida, New York, Delaware, and New Jersey). The reason for these large regional differences is not clear, but they are likely influenced by a variety of factors, including restricted access to health care services, a smaller number of nursing homes per capita, exposure to environmental risk factors, and completeness of reported data [31, 32]. Mortality rate was also found to be greater in rural areas compared to metropolitan areas, suggesting that shortages of health care resources, delays in obtaining a definitive diagnosis, or initiating preventative measures contributed significantly to the excess mortality [31, 32]. Therefore, public health interventions must take into account geographic variation in the population by increasing capacity for providing health care in rural communities, increasing awareness and access to vaccines, and improving surveillance for and management of infections in long-term care facilities.

A large proportion of deaths occurred in nursing homes and hospital settings, indicating that patients residing in these types of facilities have a higher risk of developing AD-associated pneumonia. Despite advances in care, pneumonia continues to be a frequent terminal complication among individuals with dementia [11]. Pneumonia develops in approximately 28–41% of nursing home residents having AD during an 18-month time period, causing 29–41% of AD and pneumonia-related mortalities [11, 33]. Further, AD patients have doubled the risk of developing fatal pneumonia in comparison to the general population, driven by factors including dysphagia, poor oral hygiene, decreased level of consciousness, and co-morbid conditions such as immobilization, malnutrition [11, 33–35]. Additionally, institutionalized patients are also at an increased risk of acquiring nosocomial (hospital-acquired) infections, with gram-negative bacteria and Staphylococcus aureus being two of the most common causative agents [34]. Infection control practices, training of staff members regarding the prevention of aspiration, and close monitoring of treatment in nursing homes and hospitals should be emphasized by health care policy makers. Additionally, expansion of immunizations against respiratory infections, respiratory physiotherapy, and development of specialized palliative care approaches may reduce the incidence of preventable pneumonia-related deaths in individuals with dementia.

These study results collectively demonstrate a decline in overall pneumonia and AD related mortality in older adults over the last two decades; however, substantial disparities exist by race/ethnicity, age group, and region of residence, which have implications for continued targeted preventive interventions to improve respiratory health, enhance access to healthcare for all and to continue improving the quality of long-term care for this growing and vulnerable population.

## Limitations

The present study also has some limitations. For example, since this research relies on a CDC WONDER database, there is a potential for classification bias, given that death certificates may be incomplete when it comes to indicating that pneumonia or AD was either an underlying or a contributing cause of death. In addition, although we used standardized ICD-10 codes, there can still be variability with regard to how accurately coders reported their findings, as well as how accurately medical professionals report the contributing causes of death on the death certificate. Further we have utilized the ICD-10 codes J12-J18 focusing on infectious pneumonia following the prior epidemiological studies based on similar datasets [11, 12], while we excluded J69 (aspiration pneumonia) which may result in an underestimation of the total respiratory burden in AD patients. Second, as a purely observational, population-based study, the results provide only associative evidence and do not establish a causal relationship between AD and the risk of developing pneumonia. Thus, any interpretation of these findings must be made with caution, based on the understanding that they represent correlation rather than causation. Third, due to the lack of clinical or socio-economic variables available from the database (e.g., comorbidity, vaccine use, health care access), it is difficult to fully control for confounders and to examine differences across subgroups within the sample. Fourth we are unable to account any residential migration (individuals moving to different states for retirement or care) as our analysis relied on the decedent’s state of legal residence at the time of death which might introduce mis classification regarding risk factors relevant to long-term environmental and region. Fifth, although using data through 2023 provides better temporal scope than prior studies and captures trends following the COVID-19 pandemic, the results of this study are specific to older adult populations in the U.S. and may not be generalizable to younger age groups or to older adults in other countries. Lastly, we relied on death certificate diagnoses that were largely clinical rather than pathological and our sub-analysis of the dataset indicated that only 0.65% (n = 1323) of deaths were confirmed by autopsy. As indicated by Yao et al. (2024), reliance on death certificates may underestimate pneumonia mortality compared to autopsy-confirmed cases [20].

## Conclusions

The findings of this study identify substantial demographic and regional differences in AD and pneumonia-related deaths among older Americans. The data clearly show that men, NH Whites, and those who live in rural areas and in the western U.S. had a higher disease burden than others. These demographics are at greater risk of mortality due to a lack of access to health care resources. There was a transient increase in mortality during the COVID-19 pandemic associated with increased susceptibility to acute respiratory illnesses. However, the rates normalized after the pandemic. Prevention can be improved, and disparities can be reduced through enhanced vaccination coverage, better aspiration management, earlier identification and treatment of acute respiratory illnesses, as well as by providing equal access to health care resources to all populations. Future studies need to assess the factors that increase the risk of these concurrent conditions and should develop methods to address them to reduce this preventable disease burden.

## Data Availability

The data that support the findings of this study were obtained from the publicly available Centers for Disease Control and Prevention Wide-Ranging Online Data for Epidemiologic Research (CDC WONDER) database. Aggregated datasets used in this analysis are available from the corresponding author upon reasonable request.

## References

[1] Jain S, Self W, Wunderink R, Fakhran S, Balk R, Bramley A, et al. Community-acquired pneumonia requiring hospitalization among U.S. adults. N Engl J Med. 2015. 10.1056/nejmoa1500245. 26172429 10.1056/NEJMoa1500245 PMC4728150

[2] Holland E, Jabbar A, Asghar M, Asghar N, Mistry K, Mirza M, et al. Demographic and regional trends of pneumonia mortality in the United States, 1999 to 2022. Sci Rep. 2025. 10.1038/s41598-025-94715-6. 40128337 10.1038/s41598-025-94715-6 PMC11933369

[3] Manabe T, Fujikura Y, Mizukami K, Akatsu H, Kudo K. Pneumonia-associated death in patients with dementia: A systematic review and meta-analysis. PLoS ONE. 2019. 10.1371/journal.pone.0213825. 30870526 10.1371/journal.pone.0213825 PMC6417730

[4] Rao A, Suliman A, Vuik S, Aylin P, Darzi A. Outcomes of dementia: Systematic review and meta-analysis of hospital administrative database studies. Arch Gerontol Geriatr. 2016. 10.1016/j.archger.2016.06.008. 27362971 10.1016/j.archger.2016.06.008

[5] Yao J, Liu S, Chen Q. Mortality rate of pulmonary infection in senile dementia patients: A systematic review and meta-analysis. Medicine (Baltimore). 2024. 10.1097/md.0000000000039816. 39312341 10.1097/MD.0000000000039816 PMC11419500

[6] Kalia M. Dysphagia and aspiration pneumonia in patients with Alzheimer’s disease. Metabolism. 2003;52(10 Suppl 2):36–8. 10.1016/s0026-0495(03)00300-7. 14577062 10.1016/s0026-0495(03)00300-7

[7] Bosch X, Formiga F, Cuerpo S, Torres B, Rosón B, López-Soto A. Aspiration pneumonia in old patients with dementia. Prognostic factors of mortality. Eur J Intern Med. 2012;23(8):720–6. 10.1016/j.ejim.2012.08.006. 22964260 10.1016/j.ejim.2012.08.006

[8] Better MA. Alzheimer’s disease facts and figures. Alzheimers Dement. 2023;19(4):1598–695. 10.1002/alz.13016. 36918389 10.1002/alz.13016

[9] Multiple Cause of Death Data on CDC WONDER. https://wonder.cdc.gov/mcd.html

[10] Wen J, Hu J, Zou G. Trends and differences in cardiovascular disease and Alzheimer’s disease-related mortality among older adults in the United States, 1999 to 2023: a CDC WONDER database analysis. Am Heart J Plus: Cardiol Res Pract. 2025;59:100618. 10.1016/j.ahjo.2025.100618. 10.1016/j.ahjo.2025.100618 PMC12483660 41035497

[11] Obianyo CM, Muoghalu N, Adjei EM, Njoku AN, Okoro NL, Sulaiman OB, et al. Trends and disparities in pneumonia-related mortality in the U.S. population: A nationwide analysis using the CDC WONDER data. Cureus. 2025;17(5):e83371. 10.7759/cureus.83371. 40458354 10.7759/cureus.83371 PMC12127219

[12] Ahmad F, Ahmad A, Mansoor L, Khan MK, Ahmad M. Mortality trends in heart failure and pneumonia among US adults aged 65 and older: analysis of CDC WONDER data. Cureus. 2025;17(8):e91162. 10.7759/cureus.91162. 41030754 10.7759/cureus.91162 PMC12477070

[13] Cuschieri S. The STROBE guidelines. Saudi J Anaesth. 2019;13(5):S31–4. 30930717 10.4103/sja.SJA_543_18 PMC6398292

[14] NCHS Urban-Rural Classification Scheme for Counties – PubMed. 2013. https://pubmed.ncbi.nlm.nih.gov/24776070/

[15] Age standardization of death rates: implementation of the year 2000 standard – PubMed. https://pubmed.ncbi.nlm.nih.gov/9796247/9796247

[16] Joinpoint Regression Program. https://surveillance.cancer.gov/joinpoint/

[17] Duggan M, Peng Z, Sipilä P, Lindbohm J, Chen J, Lu Y, et al. Proteomics identifies potential immunological drivers of postinfection brain atrophy and cognitive decline. Nat Aging. 2024. 10.1038/s43587-024-00682-4. 39143319 10.1038/s43587-024-00682-4 PMC11408246

[18] Ruhnke G, Coca-Perraillon M, Kitch B, Cutler D. Marked reduction in 30-day mortality among elderly patients with community-acquired pneumonia. Am J Med. 2011. 10.1016/j.amjmed.2010.08.019. 21295197 10.1016/j.amjmed.2010.08.019 PMC3064506

[19] Tejada-Vera B, Kramarow E. COVID-19 Mortality in Adults Aged 65 and Over: United States, 2020. NCHS Data Brief. 2022. 10.15620/cdc:121320. 36256450

[20] Yao J, Liu S, Chen Q. Mortality rate of pulmonary infection in senile dementia patients: a systematic review and meta-analysis. Medicine. 2024;103(38):e39816. 10.1097/MD.0000000000039816. 39312341 10.1097/MD.0000000000039816 PMC11419500

[21] Beam C, Kaneshiro C, Jang J, Reynolds C, Pedersen N, Gatz M. Differences between women and men in incidence rates of dementia and Alzheimer’s disease. J Alzheimers Dis. 2018. 10.3233/jad-180141. 30010124 10.3233/JAD-180141 PMC6226313

[22] Ono R, Sakurai T, Sugimoto T, Uchida K, Nakagawa T, Noguchi T, et al. Mortality risks and causes of death by dementia types in a Japanese cohort with dementia: NCGG-STORIES. J Alzheimers Dis. 2023. 10.3233/jad-221290. 36776074 10.3233/JAD-221290 PMC10041427

[23] Torres A, Blasi F, Dartois N, Akova M. Which individuals are at increased risk of pneumococcal disease and why? Impact of COPD, asthma, smoking, diabetes, and/or chronic heart disease on community-acquired pneumonia and invasive pneumococcal disease: Table 1. Thorax. 2015. 10.1136/thoraxjnl-2015-206780. 26219979 10.1136/thoraxjnl-2015-206780 PMC4602259

[24] Matthews K, Xu W, Gaglioti A, Holt J, Croft J, Mack D, et al. Racial and ethnic estimates of Alzheimer’s disease and related dementias in the United States (2015–2060) in adults aged ≥65 years. Alzheimers Dement. 2018. 10.1016/j.jalz.2018.06.3063. 30243772 10.1016/j.jalz.2018.06.3063 PMC6333531

[25] Chaddha J, Blaney E, Al-Salahat A, Noor A, Billion T, Chen Y, et al. Trends and disparities in Alzheimer’s disease mortality in the United States: the impact of COVID-19. NeuroSci. 2025. 10.3390/neurosci6010016. 39982268 10.3390/neurosci6010016 PMC11843863

[26] Nowalk M, Wateska A, Lin C, Schaffner W, Harrison L, Zimmerman R, et al. Racial disparities in adult pneumococcal vaccination indications and pneumococcal hospitalizations in the U.S. J Natl Med Assoc. 2019. 10.1016/j.jnma.2019.04.011. 31171344 10.1016/j.jnma.2019.04.011 PMC6888932

[27] Rajamaki B, Hartikainen S, Tolppanen A. The effect of comorbidities on survival in persons with Alzheimer’s disease: a matched cohort study. BMC Geriatr. 2021. 10.1186/s12877-021-02130-z. 33750334 10.1186/s12877-021-02130-z PMC7941944

[28] Järvinen H, Tolppanen A, Hartikainen S. Risk factors of pneumonia in persons with and without Alzheimer’s disease: a matched cohort study. BMC Geriatr. 2023. 10.1186/s12877-023-03940-z. 37038120 10.1186/s12877-023-03940-z PMC10084638

[29] Iwai-Saito K, Shobugawa Y, Aida J, Kondo K. Frailty is associated with susceptibility and severity of pneumonia in older adults (A JAGES multilevel cross-sectional study). Sci Rep. 2021. 10.1038/s41598-021-86854-3. 33846416 10.1038/s41598-021-86854-3 PMC8041848

[30] Wada H, Nakajoh K, Satoh‐Nakagawa T, Suzuki T, Ohrui T, Arai H, et al. Risk Factors of Aspiration Pneumonia in Alzheimer’s Disease Patients. Gerontology. 2001. 10.1159/000052811. 11490146 10.1159/000052811

[31] Ho J, Franco Y. The rising burden of Alzheimer’s disease mortality in rural America. SSM–Populat Health. 2022. 10.1016/j.ssmph.2022.101052. 10.1016/j.ssmph.2022.101052 PMC8886050 35242995

[32] Salerno P, Dong W, Motairek I, Makhlouf M, Saifudeen M, Moorthy S, et al. Alzheimer`s disease mortality in the United States: cross-sectional analysis of county-level socio-environmental factors. Arch Gerontol Geriatr. 2023. 10.1016/j.archger.2023.105121. 37437363 10.1016/j.archger.2023.105121

[33] Hendriks S, Smalbrugge M, Van Gageldonk-Lafeber A, Galindo-Garre F, Schipper M, Hertogh C, et al. Pneumonia, intake problems, and survival among nursing home residents with variable stages of dementia in the netherlands: results from a prospective observational study. Alzheimer Dis Ass Disord. 2017. 10.1097/wad.0000000000000171. 10.1097/WAD.0000000000000171 27849637

[34] Ai-Pin L. Etiological analysis of nosocomial infections in patients with Alzheimer’s disease and clinical treatment. Chin J Nosocomiol. 2014.

[35] Funayama M, Koreki A, Takata T, Hisamatsu T, Mizushima J, Ogino S, et al. Pneumonia risk increased by dementia-related daily living difficulties: poor oral hygiene and dysphagia as contributing factors. Am J Geriatr Psychiatry. 2023. 10.1016/j.jagp.2023.05.007. 37286391 10.1016/j.jagp.2023.05.007

